# No Sex in Science

**Published:** 1894-09

**Authors:** 


### NO SEX IN SCIENCE.

At the recent meeting of the American Dental Association, Mrs. Emma E. Chase, of St. Louis, Mo., was elected Corresponding Secretary by the unanimous vote of the members.

This is worthy of note, as showing the advance in thought in this direction since the writer introduced a resolution in this body in 1869, advocating the admission of women to the work of the profession. Now they are not only admitted upon an equality in practice, but a woman secures a responsible position in the National Association without an effort and with the applause of those present.

Mrs. Chase is a daughter of the late Professor Eames, of St. Louis.

